# Canine-Origin G3P[3] Rotavirus Strain in Child with Acute Gastroenteritis

**DOI:** 10.3201/eid1307.070239

**Published:** 2007-07

**Authors:** Simona De Grazia, Vito Martella, Giovanni M. Giammanco, Miren Iturriza Gòmara, Stefania Ramirez, Antonio Cascio, Claudia Colomba, Serenella Arista

**Affiliations:** * University of Palermo, Palermo, Italy; † University of Bari, Valenzano, Bari, Italy; ‡ Health Protection Agency, London, UK; § University of Messina, Messina, Italy

**Keywords:** human rotavirus, canine rotavirus, G3P[3] genotype, VP7, VP4, VP6, NSP4, dispatch

## Abstract

Infection by an animal-like strain of rotavirus (PA260/97) was diagnosed in a child with gastroenteritis in Palermo, Italy, in 1997. Sequence analysis of VP7, VP4, VP6, and NSP4 genes showed resemblance to a G3P[3] canine strain identified in Italy in 1996. Dogs are a potential source of human viral pathogens.

Group A rotaviruses are enteric pathogens of humans and animals. Rotaviruses usually exhibit host species restriction, although interspecies transmission or reassortment between animals and humans viruses can occur (*1*). Sequence analysis of the genes that code for the 2 outer capsid proteins VP7 and VP4, for the inner capsid protein VP6, and for the nonstructural protein NSP4 is useful for gathering epidemiologic information and tracing the origin of unusual rotavirus strains. To date, 15 VP7 genotypes (G types 1–15), 27 VP4 genotypes (P types [1]–[27]), 4 VP6 subgroup specificities (SGs I, II, I+II, and nonI/nonII), and 5 NSP4 genotypes (A–E) have been established in human and animal group A rotaviruses (*1*,*2*). Human rotaviruses usually exhibit G1, G3, G4, and G9 types in association with P[8] type, SGII specificity, and NSP4 B type; G2 rotaviruses are more often associated with P[4] type, SGI specificity, and NSP4 A type (*1*). By polyacrylamide gel electrophoresis (PAGE), most animal SGI and SGII and human SGII rotavirus strains display a “long” pattern of migration (e-type) of the 11 dsRNA genomic segments; almost all SGI human rotavirus strains possess a “short” e-type (*1*).

A number of strains with unusual VP7 and VP4 genes, regarded as animal-like, have been sporadically identified in humans and have acquired epidemiologic relevance in some geographic areas (*3*). Dogs are regarded as vectors of viral, bacterial, or parasitic zoonoses for persons of all ages, but risks for transmission of enteric viruses are almost ignored. However, early in the study of rotavirus epidemiology, symptomatic and asymptomatic infections by canine/feline-like rotavirus strains (HCR3A, HCR3B, Ro1845), characterized as G3P5A[3], long e-type and SGI, were identified in young children (*4*,*5*).

## The Study

In February 1997, rotavirus infection was diagnosed (by PAGE analysis) in a 2-year-old child hospitalized with severe acute diarrhea at the “G. Di Cristina” Children’s Hospital of Palermo. The virus, PA260/97, exhibited a long e-type and was recognized by an SG-specific monoclonal antibody (MAb) and by a VP7-specific MAb as SGI and G3 (*6*). Accordingly, strain PA260/97 displayed a genetic/antigenic constellation that is usually observed in animal-like viruses. For confirmation of the initial antigenic characterization and information about the VP4 (P) genotype, strain PA260/97 was characterized at the molecular level. By PCR genotyping of the VP7 and VP4 genes with panels of primers specific for various human G and P types (*3*,*7*,*8*), the VP7 was characterized as G3 and the VP4 was untypeable. To characterize strain PA260/97 in more detail, we determined the sequence of the VP7, VP4 (VP8*), VP6, and NSP4 genes. We also determined the VP7, NSP4, and VP6 sequences of human strain PAH101/97 (G3P[8], SGII, long e-type), detected in Palermo in the same year, as well as the sequences of the VP8*, VP6, and NSP4 genes of 2 G3P[3], SGI, long e-type strains,RV198/95, and RV52/96, isolated from dogs in Italy in 1995 and 1996, respectively (*9*).

The sequences of human strains PA260/97 and PAH101/97 and of canine strains RV198/95 and RV52/96 have been deposited in GenBank. The accession numbers are as follows: EF442738 (VP6), EF442733 (VP7), EF442735 (VP8*), and EF442741 (NSP4) for strain PA260/97; EF534715 (VP6), EF442734 (VP7), and EF534716 (NSP4) for strain PAH101/97; EF442737 (VP6), EF442736 (VP8*), and EF442739 (NSP4) for strain RV198/95; EF442742 (VP6), EF442740 (VP8*), and EF442743 (NSP4) for strain RV52/96.

**Figure 1:**
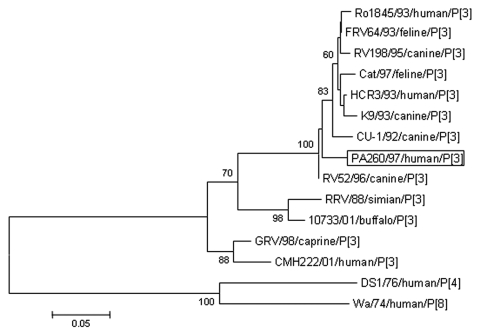
Phylogenetic analysis of the deduced amino acid sequence derived from VP4 gene of the PA260/97 human rotavirus strain and other P[3] rotavirus strains. The tree was generated by the neighbor-joining method using the ClustalW program (http://dambe.bio.uottawa.ca/dambe.asp). Scale bar indicates nucleotide substitutions (×100).

**Figure 2:**
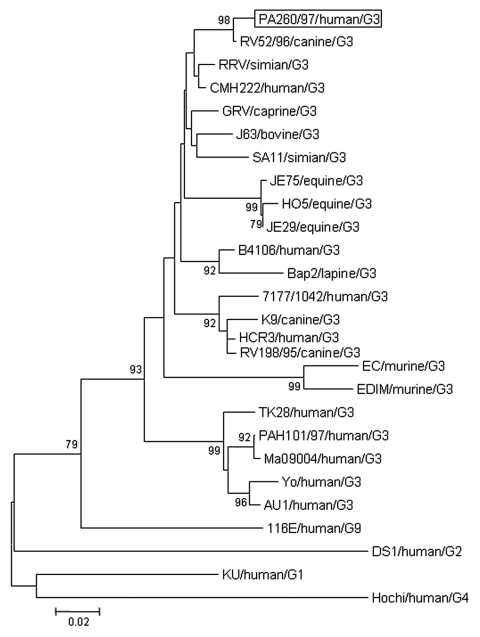
Phylogenetic analysis of deduced amino acid sequence derived from VP7 gene of the PA260/97 human rotavirus strain and other G3 rotavirus genotypes. The tree was generated by the neighbor-joining method using the ClustalW program (http://dambe.bio.uottawa.ca/dambe.asp). Scale bar indicates nucleotide substitutions (×100).

The VP8* of strain PA260/97 displayed the highest amino acid (aa) identity (98%) to the canine strain RV52/96, G3P[3], isolated in Italy in 1996 (Figure 1). Similar, the VP7 of strain PA260/97 displayed the highest identity to G3 rotaviruses, with the best match (99% nt and aa) to the canine strain RV52/96; identity to reference human G3 strains (YO, AU-1, Ma09004, TK28) and to the human G3 strain PAH101/97, isolated in Palermo in 1997, ranged from 77% to 78% nt and from 88% to 90% aa. In the phylogenetic VP7-based analysis (Figure 2), human strain PA260/97 clustered with animal G3 strains. Species-specific patterns have been demonstrated in the VP7 gene of G3 human and animal rotaviruses (*9*). These patterns are suggestive of mechanisms of host-species restriction and are useful for tracing the origin of unusual rotavirus strains.

A close genetic relationship between human strain PA260/97 and canine strain RV52/96 was also observed by comparing the genes that encode VP6 and NSP4. On the basis of MAb reactivity, strain PA260/97 was characterized as SGI and strain PAH101/97 was characterized as SGII. Sequencing of an informative fragment of the VP6 gene (*10*) showed a close genetic relationship (97% aa) between strain PA260/97 and canine strain RV52/96 and only 86% aa identity with strain PAH101/97. Sequence analyses of NSP4 enabled characterization of strains PA260/97, RV198/95, and RV52/96 into the NSP4 genotype C; strain PAH101/97 (G3P[8]) was characterized as NSP4 B genotype. The highest identity was observed between strains PA260/97 and RV52/96 (98% nt and 99% aa).

## Conclusions

Rotavirus strains with a G3P[3] combination are usually detected in cats and dogs. Despite only a few reports of rotavirus isolation from dogs with gastroenteritis, all canine strains identified thus far in the United States, Japan, and Europe (CU-1, A79-10, LSU79C-36, RS15, RV198/95, and RV52/96) display G3 and P[3] specificities (*9*). More recently, G3P[3] rotaviruses have also been identified in monkeys and goats (*1*,*11*). In contrast, detection of G3P[3] in humans is uncommon; only 4 G3P[3] strains—HCR3A, HCR3B (*4*,*12*), Ro1845 (*5*), and CMH222 (*13*)—have been reported. By sequence analysis and by RNA-RNA hybridization, strains HCR3A and Ro1845 were found to be related to canine and feline rotaviruses rather than to human G3 rotaviruses; strain CMH222 appeared to be genetically related to both simian and caprine G3P[3] rotaviruses (*13*). Genetic reassortment between human and canine rotaviruses may also have occurred. The Mexican rotavirus strain 7177-1042 bears a common human VP4 gene, P[8], in conjunction with a canine-like VP7 gene, closely related to the VP7 of canine strain RV198/95 (*14*).

Canine rotavirus infection is considered a minor disease in young dogs (pups) because it is usually mild or unapparent; however, serologic investigations have shown a high prevalence of antibodies to rotavirus in adult dogs (*15*). Previous documented examples of infections in humans by canine-like rotavirus strains have been associated with either asymptomatic (strains HCR3A and HCR3B) or symptomatic (strain Ro1845) clinical forms of disease (*4*,*5*,*12*). Strain PA260/97 in the 2-year-old child was associated with enteritis severe enough to require hospitalization. Therefore, the results of this study reinforce the hypothesis that canine-like rotaviruses may be able to not only cross the species barriers but also to induce severe disease forms in children. The lack of systematic surveillance of rotavirus infection in small animals (e.g., dogs and cats) and the fact that most rotavirus infections in such animals may go undetected hinder the ability to establish firm epidemiologic connections. In conclusion, complementing the human rotavirus surveillance programs with surveillance in animals is paramount to understanding the global ecology of rotaviruses and to identifying and characterizing interspecies transmission events and virus evolution.
